# A meta-analysis of melanoma risk in idiopathic inflammatory myopathy patients

**DOI:** 10.1007/s00393-024-01473-3

**Published:** 2024-01-29

**Authors:** Rui Luo, Dan Xia, Siyang Yu

**Affiliations:** General medicine department, The first People’s hospital of Longquanyi District Chengdu, Sichuan, China

**Keywords:** Myopathy, Dermatomyositis, Polymyositis, Skin malignancy, Risk, Myopathie, Dermatomyositis, Polymyositis, Bösartigkeit der Haut, Risiko

## Abstract

**Background:**

Idiopathic inflammatory myopathy (IIM) is a group of chronic acquired autoimmune diseases. The association between IIM and malignancies has been observed for decades. No meta-analysis has been conducted to summarize the relationship between IIM and melanoma. Herein, we specifically wanted to investigate whether IIM is associated with a higher incidence of melanoma.

**Methods:**

We searched both Chinese and English databases (CNKI, VIP, Wanfang, PubMed, Embase, Web of Science) for studies on IIM related to melanoma published up to October 2023. Two independent authors reviewed all literature to identify studies according to predefined selection criteria. Fixed effects models were applied to pool the risk. Publication bias was also evaluated and sensitivity analysis performed.

**Results:**

A total of 1660 articles were initially identified but only four cohort studies met the criteria. Thus, 4239 IIM patients were followed up. The pooled overall risk ratio/hazard ratio was 3.08 (95% confidence interval [CI] 0.79–5.37) and the standardized incidence ratio was 6.30 (95% CI 1.59–11.02).

**Conclusion:**

The present meta-analysis suggests that IIM patients are at a significantly higher risk of developing melanoma.

## Abstract (de)

**Hintergrund:**

Idiopathische inflammatorische Myopathien (IIM) stellen eine Gruppe chronischer erworbener Autoimmunerkrankungen dar. Der Zusammenhang zwischen IIM und Malignomen ist seit Jahrzehnten beobachtet worden. Bisher wurde noch keine Metaanalyse durchgeführt, um die Beziehung zwischen IIM und einem Melanom zusammenzufassen. In der vorliegenden Arbeit wurde insbesondere untersucht, ob bei Vorliegen eines Melanoms eine höhere Inzidenz für IIM besteht.

**Methoden:**

Dazu wurden sowohl chinesische als auch englischsprachige Datenbanken (CNKI, VIP und Wanfang, PubMed, Embase, Web of Science) im Hinblick auf Studien zu IIM bei Melanom durchsucht, die bis Oktober 2023 publiziert worden waren. Sämtliche Literatur wurde von 2 unabhängigen Autoren geprüft, um Studien gemäß den prädefinierten Selektionskriterien zu finden. Fixed-effects-Modelle wurden eingesetzt, um das Risiko zusammenzufassen. Auch Publikationsbias und Sensitivitätsanalysen wurden beurteilt.

**Ergebnisse:**

Initial wurden zwar 1660 Artikel gefunden, aber nur 4 Kohortenstudien erfüllten die Kriterien. Eine Nachbeobachtung war bei 4239 IIM-Patienten erfolgt. Der gepoolte Gesamt-RR/HR-Wert (relatives Risiko/Hazard Ratio) betrug 3,08 (95%-Konfidenzintervall [95%-KI]: 0,79–5,37), und der SIR-Wert (standardisierte Inzidenzratio) lag bei 6,30 (95%-KI: 1,59–11,02).

**Schlussfolgerung:**

Der vorliegenden Metaanalyse zufolge besteht bei IIM-Patienten ein signifikant höheres Risiko für das Auftreten eines Melanoms.

## Introduction

Idiopathic inflammatory myopathy (IIM) is a group of chronic acquired autoimmune diseases characterized by symmetrical muscle weakness in the proximal extremities, elevated serum myosin, and abnormal findings in electromyography and muscle biopsy. IIM includes mainly dermatomyositis (DM) and polymyositis (PM), as well as rare types such as clinically amyopathic dermatomyositis (CADM), inclusion body myositis (IBM), immune-mediated necrotizing myopathy (IMNM), and juvenile myositis (JM) [1, 2].

The association between IIM and malignancies has been observed for decades: Stertz first reported an association between DM and cancer in 1916 [3]. A population-based study in Sweden found a more than twofold risk of cancer in PM/DM compared to the general population [4]. More and more scholars subsequently found a higher risk of malignant tumors in IIM patients [5–8]. Some types of PM/DM are even proposed as a paraneoplastic skin disease. Most reviews are focused on risks in PM/DM for all types of malignancy. IIM could be combined with different types of cancers, such as lung cancer, nasopharyngeal cancer, ovarian cancer, breast cancer, stomach cancer, cervical cancer, etc. There were reviews about the relationship between IIM and site-specific cancer, such as nasopharyngeal [9] and colorectal cancer [10]. Melanoma is occasionally reported, but represents one of the most aggressive malignant tumors, dangerous for causing 90% of skin cancer mortality [11]. No meta-analysis has been conducted to summarize the relationship between IIM and melanoma. Herein, we specifically wanted to investigate whether IIM is associated with a higher incidence of melanoma.

## Methods

### Data sources and searches

Two independent authors (Luo and Xia) searched three English databases (PubMed, Embase, Web of Science) and three Chinese databases (CNKI, VIP, and Wanfang) for studies on IIM related to tumors published up to October 2023. A combination of MeSH terms and free words was used. The search strategy combined terms ‘idiopathic inflammatory myopathy,’ ‘dermatomyositis,’ ‘polymyositis,’ or ‘myositis’ with ‘cancer,’ ‘malignancy,’ ‘tumor,’ ‘tumour,’ ‘neoplasm,’ or ‘carcinoma.’ The term ‘melanoma’ was then searched in all the full texts. This meta-analysis was performed according to the guidelines specified by PRISMA [12] and registered on INPLASY under registration 10.37766/inplasy2023.11.0031.

### Study selection

The following inclusion criteria were applied: 1) written in English or Chinese; 2) type of study: cohort study reporting estimates with corresponding confidence intervals (CIs); 3) study population: patients with IIM combined with tumors; 4) outcome indicators: occurrence of tumors as one of the observed outcomes. Studies were excluded if 1) the study population was a repetitive population; 2) specific raw data indicators were unavailable; 3) the type of literature was a conference, case report, review, lecture, abstract, and so on.

### Data extraction

Two authors (Luo and Xia) independently read all literature sources and full texts. A pre-designed extraction Microsoft Excel file was used for the following information: first author, year of publication, type of study, region, period of study, number of people followed, diagnostic criteria, risk estimate such as relative risk ratio (RR), hazard ratio (HR), or standardized incidence ratio (SIR), with corresponding 95% confidence interval (95% CI). In case of any disputes, mutual discussion was organized with a third researcher.

### Assessment of research quality

The Newcastle–Ottawa Scale (NOS) was used for the quality assessment of non-randomized controlled studies. Eight items were categorized into three dimensions, which include selection, comparability, and outcome.

### Statistical analysis

The extracted data were processed using Stata software (version 16.0; Stata Corp LLC, College Station, TX, USA). The measure of interest was regarded as a continuous variable. The confidence intervals for each effect size were used with a 95% CI. We plotted a funnel plot to show the publication bias of studies: symmetry indicated no publication bias, and asymmetry publication bias requiring further examination by Begg’s correlation test and Egger’s linear regression.

The heterogeneity test was done by means of Q statistic and I^2^ test. Regarding the Q test, if *P* > 0.05, there was homogeneity among the studies; conversely, if *P* ≤ 0.05, there was heterogeneity. The size of heterogeneity was then quantitatively analyzed according to I^2^ test: if I^2^ < 50%, it indicated less heterogeneity in the included studies within the acceptable range; conversely, if I^2^ ≥ 50%, there was more heterogeneity. The corresponding effect model was selected according to the result. If *P* > 0.05 and I^2^ < 50%, the fixed effects model was used; on the contrary, if the heterogeneity was large (*P* ≤ 0.05 or I^2^ ≥ 50%), the random effects model was used for meta-analysis. The following subgroup analyses were performed if the original articles provided enough data: 1) sex, 2) age, 3) subtypes of IIM.

Sensitivity analysis was performed to assess the robustness and reliability of the combined results of meta-analysis. In this study, each single study was excluded one by one. If the effect on the combined effect size is small after eliminating a single study, it means that the results are stable and reliable; if the effect on the combined effect size is large after eliminating a single study, it means that the results are unreliable and further analysis is needed.

## Results

### Characteristics of the included studies

**Fig. 1:**
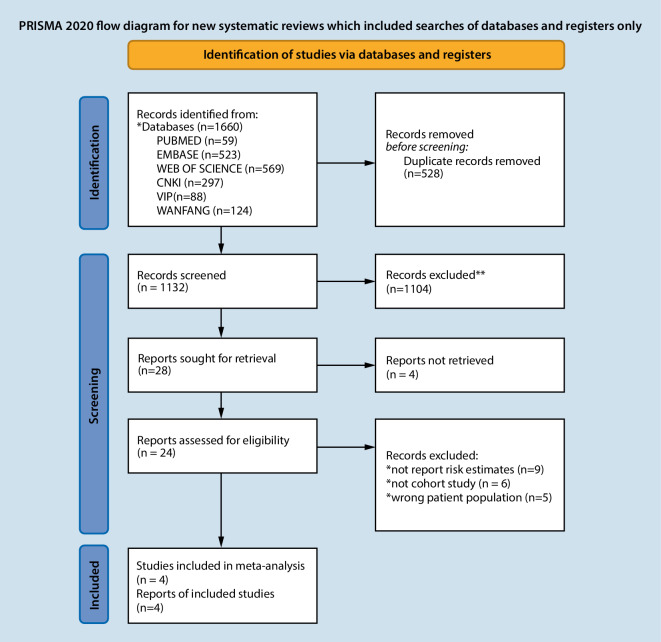
Flowchart showing study selection process

A total of 1660 papers were examined, including 59 papers in PubMed, 569 in Web of Science, 523 in Embase, 297 in CNKI, 88 in VIP, and 124 in Wanfang, with 528 removed as duplicates. By reading the title and abstract, 1104 documents were removed, and 24 documents were removed by reading the full text. Finally, 4 studies were included in the study. The literature screening flow diagram is shown in Fig. 1.

The four included papers were all cohort studies written in English, with a total sample size of 4239 IIM patients in the studies. The general information of the included studies is listed in Table 1. As shown in the table, Sigurgeirsson’s study was the earliest one, published in 1992. It followed hospitalized individuals from the Swedish National Board of Health and Welfare from 1964 through to 1983. A total of 58 cancers were diagnosed in 396 PM patients and 94 in 392 DM patients. Only one melanoma was observed after the first dermatomyositis diagnosis. Later, Stockton et al. conducted a retrospective population-based cohort study with 286 DM and 419 PM from 1982 to 1996 in Scotland. The observed number of malignant melanomas was also one, but in PM, with a standardized incidence ratio (SIR) of 3.7 (95% CI 0.1, 20.4). Chen et al. led a large sample size study of 1012 DM and 643 PM patients from 1997 to 2007 in Taiwan, China. In total, three melanoma cases were observed, one in DM with SIR 4.33 (95% CI 3.54 to 5.29) and two in PM with SIR 9.76 (95% CI 8.66 to 11.49). Dani in Sweden performed a nationwide study between 2002 and 2016. A total of 1181 patients with IIM and 6194 non-IIM comparators were followed: 14 cases of melanoma were found after IIM diagnosis, with crude incidence rate (1000 person-years) of 1.8/0.6 (IIM/non-IIM) and adjusted hazard ratio (AHR) of 3.2 (1.6–6.4).

**Table 1:** General information of the four included studies

Region	First author	Study year	Study design	Object	Criteria	IIM cases	Endpoint	Risk	LCI	UCI
Sweden	Sigurgeirsson [4]	1992	Cohort	DM/PM	ICD 7	788	RR	1.87	5.79	9.53
Scotland	Stockton [13]	2001	Cohort	DM/PM	ICD 9	705	SIR	3.7	0.1	20.4
Taiwan, China	Chen [6]	2010	Cohort	DM/PM	ICD 9	1655	SIR	7.02	1.69	12.34
Sweden	Dani [14]	2021	Cohort	IIM	ICD 10	1181	AHR	3.2	1.6	6.4

*DM* dermatomyositis, *PM* polymyositis, *IIM* idiopathic inflammatory myopathy, *ICD* international classification of diseases, *RR* relative risk, *SIR* standardized incidence ratios, *AHR* adjusted hazard ratio

The quality of the included studies was evaluated according to the Newcastle–Ottawa Quality Rating Scale. The results of the quality evaluation for each study are shown in Table 2. All four studies were of high quality, with a score of more than 5.

**Table 2:** Quality assessment of the included studies

Dimension	Item	Maximum stars	Sigurgeirsson [4]	Dani [14]	Stockton [13]	Chen [6]
Selection	Representativeness of the exposed cohort	1	1	1	1	1
	Selection of the non-exposed cohort	1	0	1	0	0
	Ascertainment of exposure	1	1	1	1	1
	Demonstration that outcome of interest was not present at start of study	1	1	1	1	1
Compatibility	Comparability of cohorts on the basis of the design or analysis	2	0	1	0	0
	Assessment of outcome	1	1	1	1	1
Outcome	Follow-up long enough for outcomes	1	1	1	1	1
	Adequacy of follow-up of cohorts	1	1	1	1	1
	total	9	6	8	6	6

### Melanoma risk

**Fig. 2:**
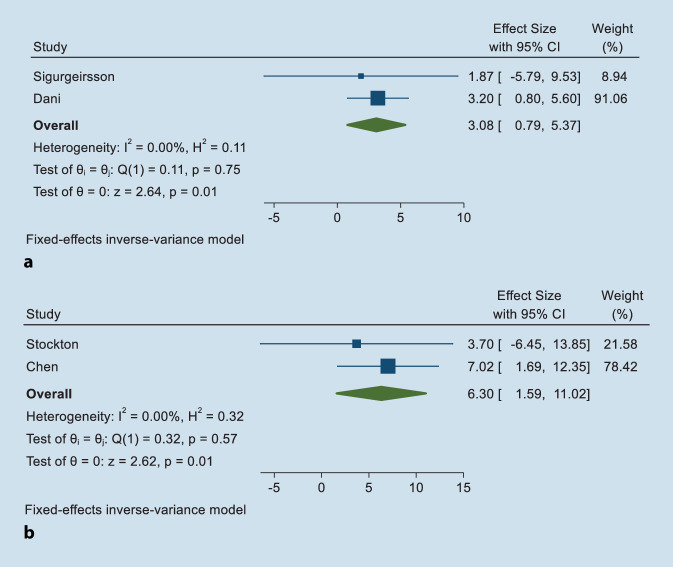
The forest plot for idiopathic inflammatory myopathy and melanoma

Four cohort studies involving 4239 IIM patients were analyzed for risk. Two studies providing RR and AHR, and another two studies providing SIR were pooled separately. The pooled overall RR/HR was 3.08 (95% CI 0.79–5.37) and the pooled overall SIR was 6.30 (95% CI 1.59–11.02), with low heterogeneity (I^2^ = 0%, *P* > 0.001) using a fixed effects model (See Fig. 2a, b). A total of 19 cases of melanoma were observed without definite age and sex information or IIM subtype, so we could not perform further corresponding subgroup analyses.

### Risk of bias assessment

The funnel plot drawn was symmetric in appearance, indicating a lack of publication bias.

## Discussion

Idiopathic inflammatory myopathy (IIM) is a group of heterogeneous inflammatory diseases characterized by symmetrical muscle weakness in the proximal extremities. The subtypes include polymyositis (PM), dermatomyositis (DM), inclusion body myositis (IBM), juvenile dermatomyositis (JDM), immune-mediated necrotizing myopathy (IMNM), and amyopathic dermatomyositis (ADM). Estimates of IIM prevalence range from 0.5 to 9.3 cases/million [15–17].

Epidemiological studies have shown that patients with DM have an increased risk of developing malignant neoplasms compared to the general population, with overall standardized incidence ratios (SIRs) ranging from 3.8 to 7.7 [18, 19]. The incidence of malignant tumors among DM patients varies widely in different literature, mostly ranging from 5% to 52%. The differences may be related to race, age, gender, and length of follow-up. In a word, the etiology and pathogenesis process are complex and still unclear. Among the connective tissue diseases, DM has been regarded as having the highest incidence of malignant tumors, with various types involving all organs. The site of malignancy has been reported as the ovary, lung, or gastrointestinal tract in Western countries and the nasopharynx in Southeast Asia, Southern China, and Northern Africa [20]. Some data show that DM is highly correlated with lung, nasopharyngeal, and colon cancer in men, and breast and ovarian cancer in women, with SIRs ranging from 8.2 to 32 [4, 7, 21, 22]. Nasopharyngeal cancer is the most prevalent in the Far East, North Africa, and the south of the country, but rare in Caucasians [23]. Chinese authors reported lung cancer to have the highest incidence in DM [24]. Population-based studies have demonstrated that PM carries a less elevated risk of cancer compared to DM [4, 21].

PM and DM are often reported in association with various malignancies, without including rare subtypes such as IBM. Thus, we did not consider IIM as a topic at first. However, as one of our final included articles followed up IIM patients, we expanded the scope from PM/DM to IIM to have a comprehensive view of myositis. In our study, the pooled overall RR/HR was 3.08 (95% CI 0.79–5.37) and the SIR was 6.30 (95% CI 1.59–11.02), which is consistent with existing research findings.

### Strengths

Melanoma has not been frequently investigated with IIM. To the best of our knowledge, this is the first meta-analysis that takes into account this most dangerous type of skin cancer. The included cohort studies are more reliable compared to case–control studies. The four studies were reported in different decades and regions, making results more representative. Table 1 showed the diagnosed criteria in each study. Moreover, the time of development of cancer was also considered, which means that all the melanoma cases were observed after the diagnosis of IIM, not before.

### Limitations

The first limitation of the study is that the number of included studies is not large. Due to the small number of 19 melanoma cases observed, there was no definite age and gender information, so we could not perform further subgroup analyses. At our initial plan, Chinese studies were searched due to the large population; however, there seemed to be a lack of high-quality cohort studies in China.

## Conclusion

In this systematic review, through meta-analysis, we present the higher risk of melanoma in IIM. More high-quality cohort studies in China are needed to gain a broader view of the association.

## Declarations

For this article no studies with human participants or animals were performed by any of the authors. All studies mentioned were in accordance with the ethical standards indicated in each case.
